# A multimodal physiological dataset for driving behaviour analysis

**DOI:** 10.1038/s41597-024-03222-2

**Published:** 2024-04-12

**Authors:** Xiaoming Tao, Dingcheng Gao, Wenqi Zhang, Tianqi Liu, Bing Du, Shanghang Zhang, Yanjun Qin

**Affiliations:** 1 Tsinghua University, Department of Electronic Engineering, Beijing, 100084 China; 2 Beijing National Research Center for Information Science and Technology (BNRist), 100084 Beijing, China; 3 University of Science and Technology Beijing, School of Computer and Communication Engineering, Beijing, 100083 China; 4 National Key Laboratory for Multimedia Information Processing, School of Computer Science, Peking University, Beijing, 100871 China

**Keywords:** Human behaviour, Neural decoding

## Abstract

Physiological signal monitoring and driver behavior analysis have gained increasing attention in both fundamental research and applied research. This study involved the analysis of driving behavior using multimodal physiological data collected from 35 participants. The data included 59-channel EEG, single-channel ECG, 4-channel EMG, single-channel GSR, and eye movement data obtained via a six-degree-of-freedom driving simulator. We categorized driving behavior into five groups: smooth driving, acceleration, deceleration, lane changing, and turning. Through extensive experiments, we confirmed that both physiological and vehicle data met the requirements. Subsequently, we developed classification models, including linear discriminant analysis (LDA), MMPNet, and EEGNet, to demonstrate the correlation between physiological data and driving behaviors. Notably, we propose a multimodal physiological dataset for analyzing driving behavior(MPDB). The MPDB dataset’s scale, accuracy, and multimodality provide unprecedented opportunities for researchers in the autonomous driving field and beyond. With this dataset, we will contribute to the field of traffic psychology and behavior.

## Background & Summary

According to the National Motor Vehicle Crash Causation Survey (NMVCCS), 94% of traffic crashes are caused by the inappropriate driver behaviour^1,2^. Some drivers may not consistently adhere to traffic regulations, potentially elevating the risk of conflicts^3^. Individual drivers exhibit distinct driving styles and levels of risk-taking propensity, influenced by factors like age and gender, affecting their perception of hazardous situations. Additionally, specific driving needs can lead to more assertive driving, potentially resulting in errors. These observations suggest a causal link between a driver’s reactions and accidents^4,5^. While there exists some research on the influence of driving state on accidents, there is a pressing need for further investigation into the impact of driving responses. Integrating human elements into traffic models offers a more comprehensive grasp of traffic modeling, control, and safety^6^. Among these considerations, comprehending the cognitive aspects of drivers and the mechanisms governing their decisions is fundamental for enhancing driver behaviour^6,7^. There remains a requirement for more human driver behavior models adaptable to a wide array of scenarios^7^. Employing physiological signals in experiments can reveal the underlying logic behind human decision-making, providing a solid foundation for modeling human driving behavior^8^.

Asymptotically homogeneous driving behaviour response dataset for complex dynamic environments will help to detect driver cognitive function based on natural driving behaviour and provide a basis for tracing the cause of accidents. Since driver cognition is an integral component of driving behaviour, driving behaviour must be studied from a cognitive and decision-making perspective, utilizing the knowledge and theory of related fields, such as psychology, physiology, engineering, and behavioural science^9–13^. Cortical beta power changes can reflect decision dynamics based on EEG. Beta power as an indicator of evidence accumulation is mainly used to study decision making^14^. To observe human behaviour, psychophysiological studies can analyse the driver’s driving state or driving intentions through physiological signals from different parts of the human body^15–20^. The development of low-power, high-precision wearable device technology has driven the study of cognition, leading to an increasing number of studies investigating the cognitive decision-making process of human driving behaviour during driving. Currently, numerous studies delve into the interpretation of EEG signals for behavioral movements. Both low-frequency EEG potentials (<3 *H*z), which are referred to as movement-related cortical potentials, and faster EEG activity, such as the sensorimotor rhythm^21^, have been related to the planning and execution of movements during both motor and imagery tasks^22–24^. Part of the literature linked anticipatory EEG signals with the contingent negative variation (CNV), a central negative deflection that can last from about 300 ms to several seconds that was previously related to sensory-motor association and expectancy^25–27^. Utilizing measurements of the CNV from low-frequency EEG, researchers have successfully decoded driver intentions in real-world driving scenarios. This research demonstrates the ability to anticipate braking and accelerating actions with a lead time of 320±200 *ms*^28–30^.

To study human responses and decision-making processes during driving tasks, researchers require rich and reproducible datasets. Table 1 summarizes the datasets from which information on drivers in survey papers as far as we know. Most existing datasets focus on predicting vehicle trajectories based on previous data or vehicle dynamics. While there have been extensive studies on vehicle-based driving behavior, attributing accidents solely to vehicle driving data proves insufficient in effectively distinguishing between driver operational errors and potential vehicle performance issues.

**Table 1:** Summary of reviewed publicly available datasets for human behaviour research in driving.

Attribute	Dataset	Tasks	Amount	Annotation	Modalities
Engineering	Pedestrian Collision Avodiance Dataset^85^	Turning	12 participants	Driver Assistance	sEMG
Computer Science	Honda Research Insitute Driving Dataset^86^	Turn Lane-change	104 hours	Driving Scene Understanding	Camera, LiDAR, GPS,IMU and CAN
	100-Car Naturalistic Driving Study (NDS) Dataset^87^	Crashes, near-crashes, other “incidents”	100-car	Driving behavior and Performance	Video, Vehicle state and kinematic sensors
	Vehicle Driving Behaviour^88^	Acceleration, normal driving, collision, turn	1032 events	Driving behaviour	Six-axis sensor
	Driving behavior Dataset^89^	Sudden Acceleration, Sudden Breaking, Sudden Turn	3 participants	Driving behaviour	Accelerometer gyroscope
	Driving Style Recognition Dataset^90^	Turning, Acceleration, Deceleration	10 participants	Driving style	GPS, Driving recorder
Psychology	multimodal distracted driving dataset^42^	No distraction, cognitive distraction, emotional distraction, sensorimotor distraction	68 participants	distracted driving	EDA, palm EDA, heart rate, breathing rate, facial signals, eye tracking
	sustained-attention driving dataset^43^	fatigue and drowsiness	27 participants	sustained-attention driving task	EEG
	multimodal driving emotions dataset^44^	Anger,Fear, Disgust,Sadness, surprise,Happniess, Neutural	40 participants	driving emotion	EEG, video, psychological data, rgb camera, infrared camera vehicle behaviour
	Collision Threat Dataset^9^	Brake	25 participants	Emergency	EEG
	Ours	Smooth driving, Acceleration, Deceleration, Lane-change, Turning	**35 participants (6052 events)**	Driving behaviour	EEG,EMG,GSR, ECG,Eye tracker

IMU = inertial measurement unit, CAN = controller area network, GPS = global positioning system, ECG = electrocardiography, EMG = electromyography, GSR = galvanic skin response, EEG = electroencephalogram, sEMG = surface electromyography.

Consequently, discerning driver behavior from vehicle performance problems presents a considerable challenge. Despite the wealth of driving data available in autonomous driving research, users exhibit hesitancy in relinquishing control over vehicles, resulting in the underutilization of this capability^31,32^. In other words, the majority of vehicles on the road remain in a non-autonomous driving state. This underscores the critical importance of human factors in driving behavior research^33,34^. In essence, conducting a comprehensive examination of driver behavior is imperative from a research standpoint. To emulate human behavior, methods like electroencephalography (EEG) and electrocardiography (ECG)^35^ play an indispensable role in quantification. Acquiring such information is more accessible compared to invasive data. Empirical evidence strongly attests to the effectiveness of these multimodal data in extracting driver behavior features.

Relying exclusively on vehicle behavior data proves inadequate in overcoming these challenges. In the actual driving process, the internal state of the driver at a certain moment is often a combination of multiple drive emotion rather than a single emotion^36,37^. For instance, when a driver is experiencing drowsiness while using the phone, a distracted state coexists, and these states may swiftly transition to an internal state of anger in response to another driver’s overtaking situation. Therefore, we cannot clearly label driving states because they are transient and unmeasurable^38–40^. However, we cannot clearly label driving states because they are transient and unmeasurable^41^. Hussain *et al*.^41^ is to establish the mapping relationship with EEG signals in both stationary and driving states. In the driving state, there is an observed increase in theta and delta waves, along with a decrease in the beta and gamma bands compared to the resting state. However, this observation does not align with the specific internal states emphasized in our article, such as anger, distraction, fatigue, etc. Despite this inconsistency, it does not contradict our claims. To some extent, it supports the notion that driving behavior can indeed be reflected in EEG signals, highlighting the complexity of the internal state during driving. We want to measure the current driver’s internalimplicit states through explicit driving behaviour data analysis besides deliberate experiment design. Mental status must be induced through well-designed experiments^42–44^. Covering all these states is difficult, so we try to reflect the intrinsic states by detecting the multimodal data to measure the driving behaviour in real time. Therefore, creating multimodal physiological signal human behaviour datasets in driving is essential for studying driver cognitive characteristics affecting driving behaviour decisions. However, to our knowledge, there are no publicly available multimodal physiological datasets of human response decisions in driving tasks. EEG signals can generate clear physiological signal analysis when we are driving, but the internal states of drivers during driving, such as distraction, anger and frustration, are relatively instantaneous changes. It is also more complex, such as angry soon into the state of frustration and so on^45^. In summary, driving behaviors are easier to annotate and map than complex emotional states. Although the internal driving state is difficult to calibrate in composite instants, we may be able to deduce the driver state from the driver’s driving behavior in future research.

Our dataset is mainly targeted at neuroscience and traffic psychology domains. Fatigue, emotion, distraction, and driving behavior are studied in this field, and it has been an important research topic in psychology, physiology, human factors engineering, and ergonomics.Our paper studies the driving behavior of drivers. At present, the common datasets in the field of driving behavior are mainly divided into four categories: 1) Driving behavior based on vehicle sensors is studied in acceleration, deceleration, turning and other aspects of driving behavior^46–50^. The data collected from vehicle sensors may be subject to inaccuracies and noise. Sensor readings can be affected by factors such as sensor calibration errors, environmental conditions, and wear and tear, leading to potential inaccuracies in the analysis of driving behavior. 2) Study the driver behavior based on the camera mounted on the vehicle^51–54^. Analysis of driving behavior may be influenced by lighting and weather conditions. Adverse weather, low light, or other visual impediments can affect the quality of images, potentially compromising the accuracy of driving behavior analysis; 3) Driving behavior is determined by the data of smart phone sensors^55–57^. Smartphones are often placed in fixed locations, such as pockets or mounts, which might not be ideal for capturing certain driving behaviors accurately. The fixed position could affect the ability to detect nuanced movements, such as steering wheel rotations or pedal usage. 4)The research of driving state based on physiological signals mainly studies the fatigue and distraction states of drivers, but there is no relevant data set that directly maps physiological signals to driver behavior^58–61^.Physiological responses to fatigue and distraction can vary significantly among individuals. What may be a reliable indicator for one driver may not hold true for another. This variability complicates the development of universal models for detecting fatigue or distraction.

We investigate the development of a dataset that directly maps physiological signals to driver behavior. Currently, there is no relevant dataset proposed. The advantages of such a dataset include: small computational requirements for physiological signal data; relatively stable physiological signal data; a clearer and more direct reflection of driving behavior with a more explicit correlation; the collection of physiological signals is not influenced by the driver’s position; simultaneously, it can improve the vehicle-human interaction interface. The application of the mapping dataset is expected to enhance the vehicle-human interaction interface. The system can intelligently respond to the driver’s physiological needs, providing a more intuitive and user-friendly interaction experience.

Here, we present a driving behaviour dataset of multimodal physiological signals for the first time. The core challenge of this dataset is how to effectively collect the driver’s reaction decisions and behaviour during driving. Therefore, the core work is to obtain multimodal driving behaviour datasets and analyse different human driving behaviours by designing experiments based on Event-Related Desynchronization/Synchronization (ERD/ERS) paradigm^62–64^ and combining data from physiological signals. We did not utilise Event-Related Potentials (ERPs) as an EEG experimental paradigm in our experiments. The experimental design of ERPs requires a single stimulus such as flashing brake lights^65^. Specific stimuli in ERP experiments cannot be reproduced in dynamic driving scenarios. Our experimental paradigm is Event-Related Desynchronization/Synchronization (ERD/ERS). Cortical beta power changes can reflect decision dynamics based on EEG. Beta power as an indicator of evidence accumulation is mainly used to study decision making^14^.

First, we design four different driving tasks in the same scene. Then, we conducted driving experiments on the 51 WORLD driving simulator. The conventional scalp EEG caps for the EEGs we used is a non-invasive method to record scalp voltage over time. The electroencephalography (EEG), electrocardiogram (ECG) and electromyogram (EMG) device model is Neuracle, and the eye tracker model is Tobii Glasses 2. The driving simulation software synchronizes all the equipment by sending time stamps to the trigger box. Finally, data preprocessing is mainly carried out using the EEGLAB^66^ plug-in of MATLAB. The preprocessed data are analysed in the time-frequency domain by MATLAB, and feature downscaling and classification are carried out using methods such as linear discriminant analysis (LDA).

## Methods

### Participants

The content and procedures of this study were noticed and approved by the Medical Ethics Committee of Tsinghua University(the approval number: 20230007). Thirty-five voluntary participants (age range = 20–60 years old, average age = 25.06 years old, SD = 7.90), who were students or faculty members at Tsinghua University were recruited to participate in 150-minute event-related driving tasks, including 26 males and 9 females. The participants must have a driving licence of the People’s Republic of China above grade C (including grade C) and had at least one year of driving experience (driving experience range = 1–20 years, average driving experience = 3.03 years, SD = 3.68). Participants were required to ensure adequate rest (sleep no less than 8 hours) before the experiment, and to not stay up late the night before the experiment (sleep no later than 12:00 p.m.) to reduce the impact of noise on EEG signals. The participants had no hair perms or drug use history within three months and did not take any excitatory substances within 48 hours before the experiment, including but not limited to tranquilizers, tranquilizers, alcohol, coffee, tea and cigarettes. All participants had no mental disorders and were required to complete the experiment according to their actual driving style. A pretest will be conducted before the experiment to ensure that all participants understand the experimental task and that no participants have physiological discomfort due to the simulated environment of the experiment. All participants were informed of the experimental requirements before participating in the experiment and the economic reward for an experiment was 90–120 yuan per hour. Each participant completed all five types of driving events, spread across seven or eight sets of driving tasks.

Desired sample size was based on G*Power analysis. We used F-tests, ‘ANOVA: Repeated measures, between factors’ to compute required sample size. We set *f* = 0.5, *α* = 0.05, Power = 0.8, Number of groups = 5, Number of measurements = 40, G*Power produced a recommended sample size of 30 participants. Among them, *α* and Power are determined based on the basic theory of mathematical statistics^67^. The number of measurements is the average number of events per subject. The number of groups correspond to the categories of the events. Effect size *f* is determined based on large effect with values ranging from 0.5 to 0.8^68–71^.

### Experimental environment

**Fig. 1:**
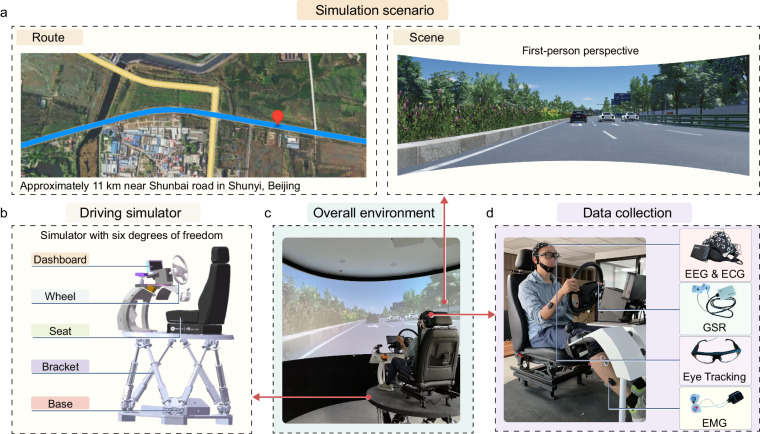
Experimental setup of multimodal human driver factor data collection. (**a**) A brief sketch of the overall simulation scenario. (**b**) Structural diagram of the simulator with six degrees of freedom. (**c**) Overall environment. All participants completed the experiment in the same environment. (**d**) Data collection. The multimodal physiological data to be collected are shown in Fig. 1, and the use of the relevant portraits was authorized by the participants.

The experimental environment is mainly composed of a driving simulator and a circular curtain, as shown in Fig. 1a. The driving simulator contains a six-degree-of-freedom motion platform and a control platform. The carrying capacity of the six-degree-of-freedom motion platform reaches 500 kg, and during the experiment, the platform can achieve the functions of translation and rotation, in which the maximum stroke of translation motion can reach 400 mm, the maximum acceleration can reach ±0.7 g, and the maximum speed can reach 400 mm/s. The maximum amplitude of rotation motion can reach ±23°, the maximum acceleration is ±500°/*s*^2^, and the maximum speed is 40°/*s*. The steering wheel of the control platform adopts the real car disk surface, which is directly driven by a servo motor. The strength of the steering wheel is linearly adjustable and the peak torque can reach 28.65 Nm. The servo motor communicates with the control system through a coding device, the control system contains a servo driver and motion control card, the servo driver is responsible for driving the servo motor, the motion control card is responsible for the interaction between the torque signal and the steering wheel signal with the computer, and the two communicate through an encoder signal.

The scene used in the experiment is provided by 51world, which simulates the actual road in Beijing and is projected onto the circular curtain. The actual road section, which is approximately 11 km near Shunbai road in Shunyi, Beijing, is intercepted as the simulation scene to better restore the actual driving environment. The experimental route is shown in Fig. 1b. The road scene is relatively rich, including multiple right angle curves, four lane straight urban roads, two lane straight urban roads, etc. The refresh rate of the scene frame is approximately 60 fps, and the simulation image is shown in Fig. 1c. The main vehicle is controlled by the driving simulator operated by the participants. The static elements and the opponent vehicle are designed to realize various events according to the location of the main vehicle. The participants needed to pay attention to the road conditions at all times and take measures for the events. A variety of physiological signal acquisition devices synchronously recorded the physiological data changes before and after the participants engaged in driving behaviour.

### Experimental paradigm

**Fig. 2:**
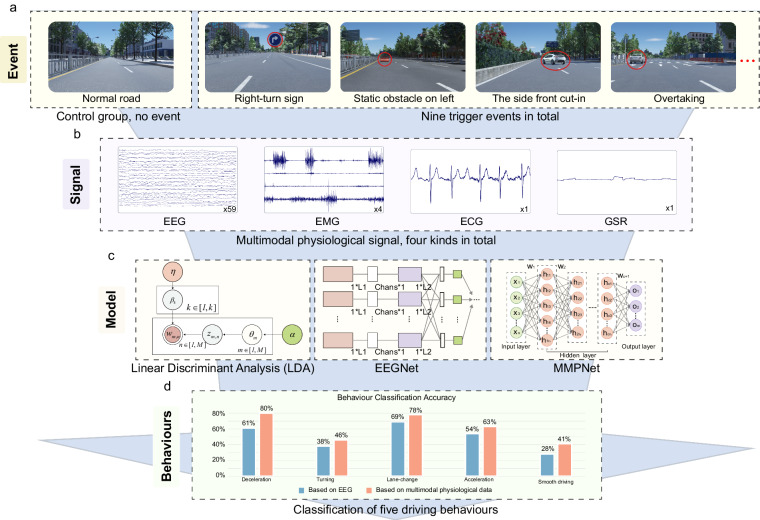
The overall design framework of the experiment. (**a**) Trigger event. (**b**) Signal input. (**c**) Model selection. (**d**) Behavioural output. The overall idea is to induce different driving behaviour of human drivers through specific events and synchronously collect multimodal physiological signals, and then use models, such as LDA and EEGNet, to classify multiple driving behaviours.

**Fig. 3:**
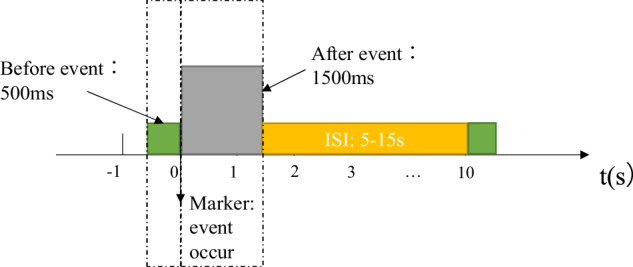
Marker used to mark the event occurrence point. With reference to the event related potential (ERP) paradigm, 500 ms before the event and 1500 ms after the event were intercepted to analyse the relationship between physiological signals and driving behaviours. To reduce mutual interference between events, there is a time interval between two events.

**Fig. 4:**
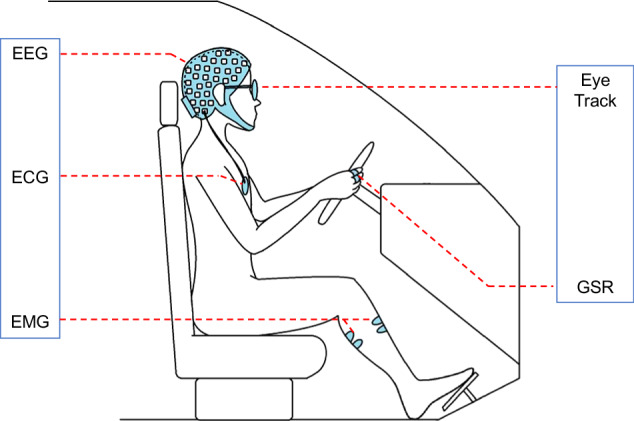
Location of all sensors. The EEG cap, EMG electrodes, GSR electrodes, ECG electrodes, and eye tracker are placed in the positions as shown.

The experiment adopted the event-related behaviour response paradigm to model various driving behaviours using event-related multimodal physiological signals, as shown in Fig. 2. This experimental design covers a relatively comprehensive range of basic driving behaviours, which were carried out by the subjects participating in the experiment, and the driver’s multimodal physiological data were collected synchronously. The exact location of the sensors used in the experiment is shown in Fig. 4. The driving behaviours of our experiment are relatively rich, and the collected physiological data have a higher dimension, that is, the data may contain more information. By designing specific events to induce different driving behaviours, the participants were required to respond to the events to record the multimodal physiological data when the participants engaged in different driving behaviours. Specifically, the driving behaviours to be induced were divided into five categories: smooth driving (control group), acceleration, deceleration, lane-change and turning. The selection of these five types of behaviours takes into account the driver’s basic operations, namely, pedal-based operations and steering wheel-based operations. Smooth driving is the control group. Pedal-based operations include acceleration and deceleration, and steering wheel-based operations include lane changing and turning. Various behaviours are triggered by different events. To ensure the balance of the number of samples, the number of samples corresponding to different driving behaviours should be the same as similar as possible. As shown in Table 2, smooth driving is triggered by normal straight-line driving; acceleration is triggered by overtaking and congestion relief; deceleration is triggered by the sudden braking of the vehicle in front, the sudden lane change of the vehicle in front of the side, and the pedestrian crossing the road; lane-change is triggered by static obstacles in front; turning is triggered by right angle left turn indication and right angle right turn indication. When the trigger event occurs, the time is marked as the marker of event-related potential (ERP) to facilitate data segmentation and research during data analysis, as shown in Fig. 3. Different driving behaviours were divided into four different cases (considering the actual setting of the case, the two behaviours of smooth driving and turning were in the same case). Each participant completed eight groups of experiments. Notably, the time interval between two adjacent events was 5–15 s, and the participants were required to manoeuvre the vehicle back to the original lane and resume normal driving within this period of time. In each experiment, the participant needs to drive the entire journey. The duration of all driving tasks was approximately 90 min, and a rest time was interspersed between them to ensure that the participants do not enter a fatigued driving state. All the operating procedures of the participants and the physiological data during the operation were synchronously recorded.

**Table 2:** Correspondence between driving behaviour and triggering events.

Driving behaviour	Corresponding events	The number for marker	Expected number of samples per participant
Smooth driving	Normal straight line driving	133	18
Acceleration	Overtaking	135	24
	Congestion relief	143	24
Deceleration	The front emergency brake	139	16
	The side front cut-in	141	16
	Pedestrian crossing	145	16
Lane-change	Static obstacle on right	131	24
	Static obstacle on left	129	24
Turning	Left-turn sign	125	18
	Right-turn sign	127	18

The setting conditions and time mark positions corresponding to various trigger events were different, and are summarized as follows:

1. Smooth driving: Smooth driving in our experimental design is relative to other mark with event triggering as a control, which refers to the state when no event occurs and the vehicle is travelling on an empty straight road, as a control group. When the main vehicle drives in the normal straight line driving area which is defined in the map design, the time mark is made.
2. Acceleration: The acceleration behaviour has two types of trigger conditions. The first is the overtaking scenario, where the main vehicle will encounter a slower moving vehicle in front of it (speed set to 5 m/s and the driver needs to complete the overtaking action after noticing the slow moving vehicle. The time marker is set when the opponent car starts to drive slowly, as shown in Fig. 5d.

**Fig. 5:**
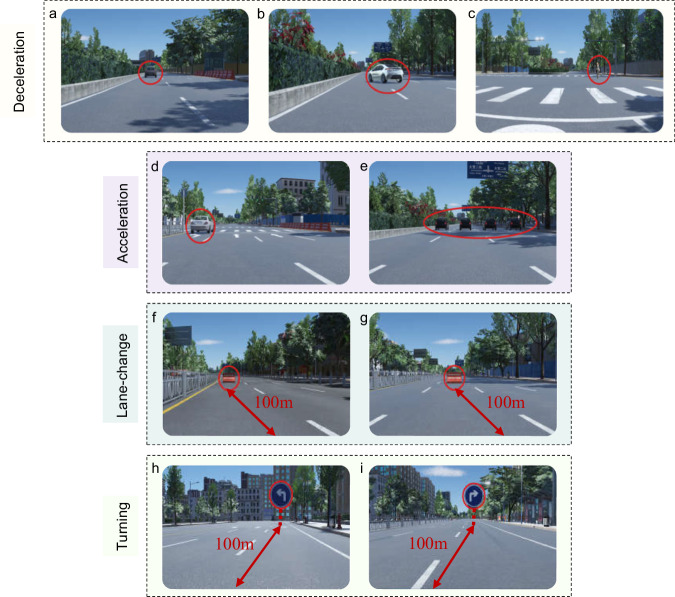
Event setup used to induce human driving behaviours, including acceleration, deceleration, lane-change and turning. (**a**)The front emergency brake. (**b**)The side front cut-in. (**c**) Pedestrian crossing. (**d**) Overtaking. (**e**) Congestion relief. (**f**) Static obstacle on left. (**g**) Static obstacle on right. (**h**) Left-turn sign. (**i**) Right-turn sign.
  The second is the congestion relief scenario, in which the main car encounters multiple cars blocking the road (the speed was set to 5 m/s simulating a congestion scenario). And the opponent cars will rush out at a fast speed to simulate congestion relief after driving slowly for 50 m. At this time, the driver needs to accelerate because they were asked to keep the normal speed at no less than 60 km/h, and the time marker is set at the time when multiple opponent cars accelerate to rush out, as shown in Fig. 5e.
3. Deceleration: There are three scenarios that prompt deceleration. The first involves an emergency braking situation with the vehicle in front. As the main vehicle follows, both vehicles maintain a consistent speed. However, after traveling 50 meters, the vehicle in front suddenly applies the brakes. This leads to a rapid reduction in speed, potentially reaching 0 km/h. Participants were instructed to maintain a speed of no less than 60 km/h and a following distance of only 100 meters. Consequently, they must decelerate swiftly upon encountering such a situation to avert a rear-end collision. The time mark is located when the opponent car in front decelerates to 0, as shown in Fig. 5a.
  The second is the situation of a sharp lane change of the vehicle on the side in front. When the main vehicle drives in accordance with the specified lane, there will be a opponent vehicle driving at 0.7 times the speed of the main vehicle on the side in front when driving to a fixed position, and the opponent vehicle will urgently change the lane to the lane where the main vehicle is located after driving for 50 m. The driver needs to slow down after discovering this change to prevent a rear end collision. The time mark is located in the sharp lane change of the vehicle on the side in front, as shown in Fig. 5b.
  The third is the situation of pedestrians crossing the road. When the main vehicle reaches a fixed position, pedestrians cross the road at a speed of 5 m/s in front of it. The driver needs to respond in time and slow down to prevent hitting pedestrians. The time mark is when pedestrians begin to cross the road, as shown in Fig. 5c.
4. Lane-change: There are two types of trigger conditions for lane change: both are static obstacles, and the difference is that there are two kinds of static obstacles blocking the road ahead. To guide the driver to achieve left lane change and right lane change, the driver needs to complete the corresponding lane change behaviour after observing the static obstacles. The case design delineates the lane change area, the starting point of which is approximately 100 m from the static obstacles. This position is the position where the relatively stable obstacles found through the test appear in the simulation screen, and the time mark is when the main vehicle drives into the area, as shown in Fig. 5f,g.
5. Turning: There are two kinds of trigger conditions for turning behaviour, road turn signs, which are divided into left-turn signs and right-turn signs. They guide the driver to turn left and right to ensure that the main vehicle runs according to the specified route. The driver needs to complete the corresponding turning behaviour after observing the sign. In the case design, the turning area is delimited, which is similar to the lane-change area. The starting point of the turning area is approximately 100 m away from the indicated road sign. This position is the position where the relatively stable indicated road sign found through the test appears in the simulation screen. When the main vehicle drives into the area, the time mark is recorded, as shown in Fig. 5h,i.

### Data synchronization

During the experiment, multimodal physiological data were recorded synchronously. Synchronization is achieved by the synchronization trigger box provided by Neuracle in China, which is called the NeuSen TB series multiparameter synchronizer. When the driving simulator data collection computer receives a specific stimulus, it sends a time marker to the physiological data side through the synchronization trigger box, and all these time markers are recorded in a “bdf” file. Through this synchronization mark, the physiological data corresponding to driving behaviour are aligned to realize synchronization of data recording.

### EEG and ECG signals

The EEG signals were collected by the physiological data collection system called the NeuSen W Series Wireless EEG Acquisition System, which is provided by Neuracle. The system includes an EEG cap with 64 electrodes, 59 of which were used to collect the EEG signals for subsequent data analysis. The cap was worn on the participant’s head to record the EEG signals, and the EEG signals were recorded in a “bdf” file with a sampling frequency of 1000 Hz.

**Fig. 6:**
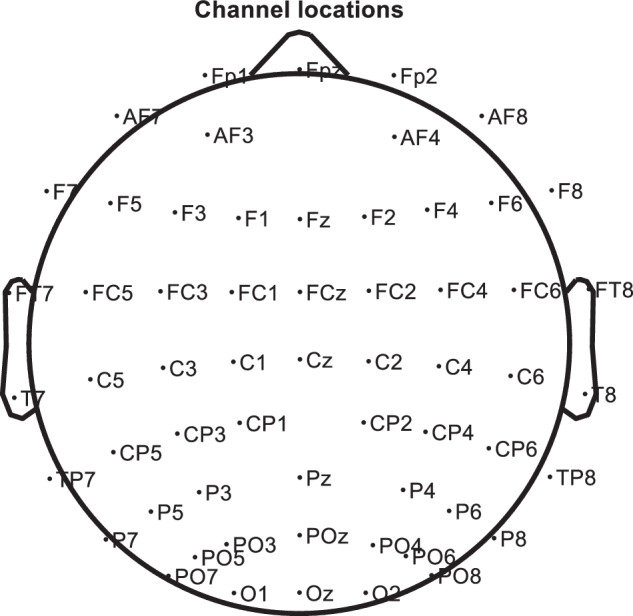
Location of EEG electrodes. 59 electrode locations shown.

The electrode position distribution of EEG acquisition instruments has a unified customary standard, arranged according to the international 10–20 electrode system developed by the EEG Society^72^. The electrode distribution of this system is shown in Fig. 6, where the electrodes are named by letters and numbers, with letters representing the cortical area where the electrodes are located, odd numbers representing the left brain part, and even numbers representing the right brain part.

The ECG signal is also collected through the Neuracle system since one of the electrodes in the cap is for ECG recording. The ECG electrode can be applied to the participant’s chest near the heart through an electrode patch to achieve ECG data collection. The sampling frequency was also 1000 Hz and the data were recorded in a “bdf” file.

### EMG signals

The EMG signals were acquired by the NeuSen WM Series Wireless EMG Acquisition System provided by Neuracle. The acquisition module was pasted on the tibialis anterior muscle, gastrocnemius muscle of the right leg and brachioradialis muscle of both arms, for these muscles are involved in a person’s driving behaviour during driving. The tibialis anterior and gastrocnemius muscles of the right leg are mainly involved in the braking and throttle operation of the legs^73,74^, while the brachioradialis muscles of the arms are involved in the control of the steering wheel^75,76^. The acquisition module communicates with the device base station through Bluetooth, and the base station and the acquisition computer realize multimodule synchronous acquisition through a wired network connection. The sampling frequency was also 1000 Hz and data were recorded in a “bdf” file.

### GSR signal

The GSR signal was collected by the NeuSen W GSR Series Wireless GSR Acquisition System provided by Neuracle, and the collection electrode was adhered to the belly of the participants’ left index finger and middle finger. The sampling frequency was also 1000 Hz and data were recorded in a “bdf” file.

### Eye track signals

The oculomotor signal was collected by Tobbi Glasses 2, which can be configured according to the participant’s desired visual correction, thus ensuring that the participant has normal or corrected vision at the time of the experiment. The participant wore an oculomotor, and the raw data collected by the oculomotor contained time-stamped information, visual fall point, eye position, pupil diameter, etc. The sampling frequency was 100 Hz and data were recorded in a “ttgp” file.

## Data Records

### Data recording and storage

In this section, we will clarify the storage organization of MPDB dataset, which is publicly accessible in Figshare, including raw dataset^77^, preprocessed dataset^78^, and eye tracking dataset^79^. The raw dataset contains physiological data of 35 subjects driving for 2 hours each, and the preprocessed dataset contains physiological data samples of the driving behaviour of 35 subjects.

#### Raw dataset storage

The organization of the dataset folder is shown in Table 3. The directory of each subject includes data from four experiments, and each experiment corresponds to different behaviors, namely, deceleration, acceleration, turning, and lane change. Therefore, the {event} field of the filename should be replaced with {brake, turn, throttle, change} when obtaining the data of different behaviors, as shown in the “Raw Dataset” section in Table 3. You can frame and prerprocess the raw data according to your own needs.

**Table 3:** The file names of the raw dataset and the preprocessed dataset.

Subject	Filename						
	Raw Dataset			Preprocessed Dataset			
	EEG&ECG	EMG	GSR	EEG	EMG	GSR	ECG
1	1_{event}.set	1_{event}.set	1_{event}.set	EEG_1.set	EMG_1.set	GSR_1.set	ECG_1.set
2	2_{event}.set	2_{event}.set	2_{event}.set	EEG_2.set	EMG_2.set	GSR_2.set	ECG_2.set
3	3_{event}.set	3_{event}.set	3_{event}.set	EEG_3.set	EMG_3.set	GSR_3.set	ECG_3.set
4	4_{event}.set	4_{event}.set	4_{event}.set	EEG_4.set	EMG_4.set	GSR_4.set	ECG_4.set
5	5_{event}.set	5_{event}.set	5_{event}.set	EEG_5.set	EMG_5.set	GSR_5.set	ECG_5.set
6	6_{event}.set	6_{event}.set	6_{event}.set	EEG_6.set	EMG_6.set	GSR_6.set	ECG_6.set
7	7_{event}.set	7_{event}.set	7_{event}.set	EEG_7.set	EMG_7.set	GSR_7.set	ECG_7.set
8	8_{event}.set	8_{event}.set	8_{event}.set	EEG_8.set	EMG_8.set	GSR_8.set	ECG_8.set
9	9_{event}.set	9_{event}.set	9_{event}.set	EEG_9.set	EMG_9.set	GSR_9.set	ECG_9.set
10	10_{event}.set	10_{event}.set	10_{event}.set	EEG_10.set	EMG_10.set	GSR_10.set	ECG_10.set
11	11_{event}.set	11_{event}.set	11_{event}.set	EEG_11.set	EMG_11.set	GSR_11.set	ECG_11.set
12	12_{event}.set	12_{event}.set	12_{event}.set	EEG_12.set	EMG_12.set	GSR_12.set	ECG_12.set
13	13_{event}.set	13_{event}.set	13_{event}.set	EEG_13.set	EMG_13.set	GSR_13.set	ECG_13.set
14	14_{event}.set	14_{event}.set	14_{event}.set	EEG_14.set	EMG_14.set	GSR_14.set	ECG_14.set
15	15_{event}.set	15_{event}.set	15_{event}.set	EEG_15.set	EMG_15.set	GSR_15.set	ECG_15.set
16	16_{event}.set	16_{event}.set	16_{event}.set	EEG_16.set	EMG_16.set	GSR_16.set	ECG_16.set
17	17_{event}.set	17_{event}.set	17_{event}.set	EEG_17.set	EMG_17.set	GSR_17.set	ECG_17.set
18	18_{event}.set	18_{event}.set	18_{event}.set	EEG_18.set	EMG_18.set	GSR_18.set	ECG_18.set
19	19_{event}.set	19_{event}.set	19_{event}.set	EEG_19.set	EMG_19.set	GSR_19.set	ECG_19.set
20	20_{event}.set	20_{event}.set	20_{event}.set	EEG_20.set	EMG_20.set	GSR_20.set	ECG_20.set
21	21_{event}.set	21_{event}.set	21_{event}.set	EEG_21.set	EMG_21.set	GSR_21.set	ECG_21.set
22	22_{event}.set	22_{event}.set	22_{event}.set	EEG_22.set	EMG_22.set	GSR_22.set	ECG_22.set
23	23_{event}.set	23_{event}.set	23_{event}.set	EEG_23.set	EMG_23.set	GSR_23.set	ECG_23.set
24	24_{event}.set	24_{event}.set	24_{event}.set	EEG_24.set	EMG_24.set	GSR_24.set	ECG_24.set
25	25_{event}.set	25_{event}.set	25_{event}.set	EEG_25.set	EMG_25.set	GSR_25.set	ECG_25.set
26	26_{event}.set	26_{event}.set	26_{event}.set	EEG_26.set	EMG_26.set	GSR_26.set	ECG_26.set
27	27_{event}.set	27_{event}.set	27_{event}.set	EEG_27.set	EMG_27.set	GSR_27.set	ECG_27.set
28	28_{event}.set	28_{event}.set	28_{event}.set	EEG_28.set	EMG_28.set	GSR_28.set	ECG_28.set
29	29_{event}.set	29_{event}.set	29_{event}.set	EEG_29.set	EMG_29.set	GSR_29.set	ECG_29.set
30	30_{event}.set	30_{event}.set	30_{event}.set	EEG_30.set	EMG_30.set	GSR_30.set	ECG_30.set
31	31_{event}.set	31_{event}.set	31_{event}.set	EEG_31.set	EMG_31.set	GSR_31.set	ECG_31.set
32	32_{event}.set	32_{event}.set	32_{event}.set	EEG_32.set	EMG_32.set	GSR_32.set	ECG_32.set
33	33_{event}.set	33_{event}.set	33_{event}.set	EEG_33.set	EMG_33.set	GSR_33.set	ECG_33.set
34	34_{event}.set	34_{event}.set	34_{event}.set	EEG_34.set	EMG_34.set	GSR_34.set	ECG_34.set
35	35_{event}.set	35_{event}.set	35_{event}.set	EEG_35.set	EMG_35.set	GSR_35.set	ECG_35.set

#### Preprocessed dataset storage

After all, we have uploaded the raw dataset and the preprocessed dataset to the publicly accessible repository of figshare. In the preprocessed dataset, the behaviour samples of each subject are combined into a file. These samples include five types of behaviors, and the event types in each behaviour are shown in the Table 2. Readers can find these event types in “EEG.events.type” of the EEG structure when using MATLAB to read the datasets. Since EEG and ECG were collected through the same wireless transmission device, the two were separated during preprocessing and the raw data were organized by subject number. For each modality, all behavioural data from each subject were combined into one data file and named using the corresponding modality and subject number, as shown in the “Preprocessed Dataset” section in Table 3.

## Technical Validation

In this section, we prove the validity of the dataset. According to the convention of physiological data validation and the experiment, we consider the following three aspects^80^: whether physiological data can be used, whether vehicle parameters are correct, and whether there is a correlation between physiological data and driving behaviours. To this end, technical validation includes quality validation of physiological variables and vehicle parameters and correlation analysis of physiological variables and behaviours. In the last part, several classification models are used to effectively prove the validity of the data^81^.

### Physiological data validation

This part explains the availability and standard of the physiological data in this dataset. Each kind of physiological data was preprocessed before use, and the preprocessing method conformed to the specifications of physiological data preprocessing, which is described in detail below.

The technical validity of the physiological dataset is highly related to the equipment and acquisition specification process of the experiment. For example, whether the impedance is in a reasonable range, whether the data processing method is standardized, and so on. Table 4 demonstrates the parameters of the experimental equipment, and it is evident that its accuracy meets the needs of physiological signal acquisition.

**Table 4:** Main parameters of the data acquisition equipments.

Device	Parameters
EEG	CMRR:≥120 dB
EMG	base noise: < 1uVrms
GSR	CMRR:100 dB

For each physiological variable collected in the experiment, we drew their waveforms as time functions for verification. The overall results of each type of data for each subject are shown in Figs. 10–13.

#### EEG validation

The EEG data for this dataset include 59 channels, the sampling frequency is 1 kHz, and the sample length of each behaviour is 2 s, so each frame contains 2000*59 sampling points.

##### Impedance validation

EEG signals were collected by a head-worn device, so the hair of the participants affects the quality of the signal. In the preparation stage of the experiment, it is necessary to inject conductive paste into each electrode to ensure reliable contact with the scalp. Excessive impedance will reduce the quality of the signal and cause greater noise. During the test, we ensured that the impedance of each electrode was lower than 20 k Ω, and each experiment was carried out after confirming that the data waveform was normal.

##### Data preprocessing validation

We preprocessed the raw EEG signal to suppress noise, remove artifacts, and extract useful information. The preprocessing steps mainly include bandpass filtering, enframing and artefact removal. According to the useful frequency band of the EEG signal, an IIR bandpass filter with a [0.5 Hz 40 Hz] pass band is used for filtering.

**Fig. 7:**
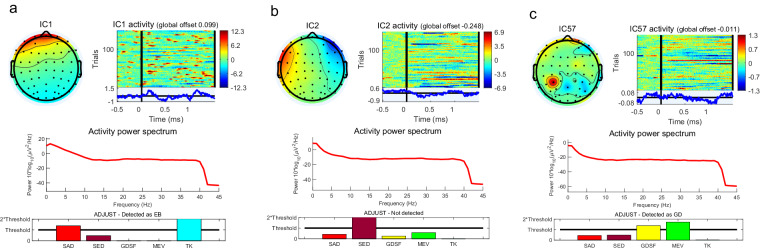
Three types of typical artifacts: (**a**) Eye blinks; (**b**) Eye movement; (**c**) Abnormal electrode artifact.

Take the corresponding time of each mark as the centre, we can select the [−0.5 s, 1.5 s] interval as the corresponding behaviour samples for the filtered data, which was based on the data partitioning method used in event-related potential experiments^73^. We provided this as a reference for technical validation, rather than a mandatory segmentation. Finally, ICA decomposition is performed on these behaviour samples to remove artefacts. EEGLAB will determine whether each independent component is a useful signal or a artifact based on several objective indicators. We make a comprehensive judgment based on the recommendations of EEGLAB and typical artifact paradigms, including obvious eye movement artifacts, muscle movement artifacts and abnormal electrode artifacts^82^, as shown in Fig. 7. The EEG waveform is shown in Fig. 10. It contains 59 channels of valid data from five categories of driving behaviours. From the waveform, it can be observed that the EEG signals corresponding to each category of events meet the requirements, with no significant anomalies in the samples.

##### Physiological structure

**Fig. 8:**
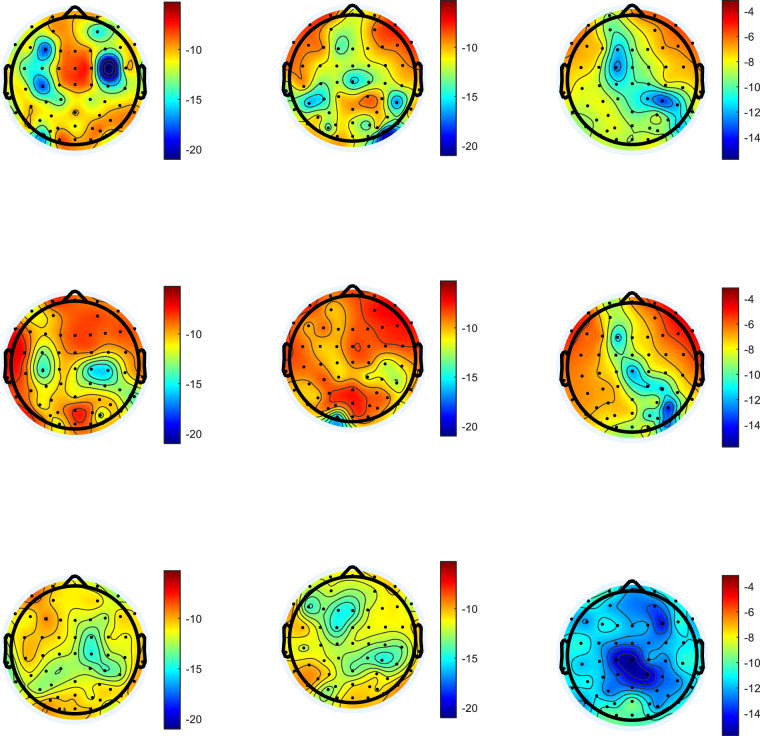
Power spectral density of three braking events.

In order to analyze the physiological components of driving behaviors embodied in the EEG signals, we extracted the EEG time-frequency domain features of each driving behavior by using the short-time Fourier transform (STFT), and selected 500 ms before the event, 200 ms after the event and 1500 ms after the event as the three observed moments, as shown in Fig. 8. It can be seen from the figure that among the three events that caused the braking behavior, the driver’s EEG signal showed an increase in power at 200 ms, mainly in the parietal and temporal lobes.

##### Statistical property validation

**Fig. 9:**
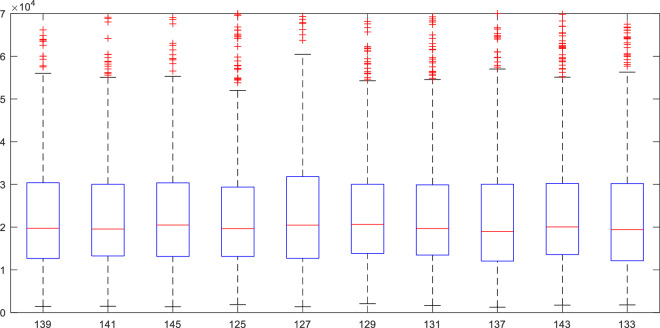
Box plots of EEG power spectra for different events.

We analyzed the statistical properties of the EEG data. The EEG power spectral densities under each event are shown in Table 5, which lists the average EEG power spectral densities PSD (dB/Hz) as well as the mean and standard deviation for all participants under each stimulus condition. The boxplot of EEG power spectra for different events is depicted in Fig. 9, which indicates that the power distribution of each group is essentially identical.

**Table 5:** The statistical properties of the EEG power spectral densities.

Driving behaviour	Stim Type	Sample Size	EEG PSD	
			Mean	Std
Deceleration	139	430	7.0314	0.3113
	141	480	7.0792	0.3026
	145	415	7.016	0.309
Turning	125	499	7.096	0.2849
	127	503	7.0995	0.3007
Lane-change	129	864	7.3345	0.2727
	131	901	7.3527	0.2919
Acceleration	137	768	7.2833	0.3112
	143	734	7.2636	0.2998
Smooth driving	133	950	7.3757	0.3233

#### EMG, GSR and ECG validation

The dataset also includes EMG, ECG and GSR signals, which can be regarded as discrete time series. The EMG signal consists of four channels with a sampling rate of 1 kHz, which were collected on two arms and the right leg. The GSR and ECG both contain one channel with a sampling rate of 1 kHz. These three signals were all preprocessed to suppress noise and extract useful information. The preprocessing steps include bandpass filtering and removing bad samples. The range of bandpass filtering is selected as^15,83^ Hz for EMG, [0.01, 200] Hz for ECG, and [0.5, 100] Hz for GSR. The waveforms of the extracted valid EMG, GSR, and ECG data samples for each test are shown in Figs. 11–13.

**Fig. 10:**
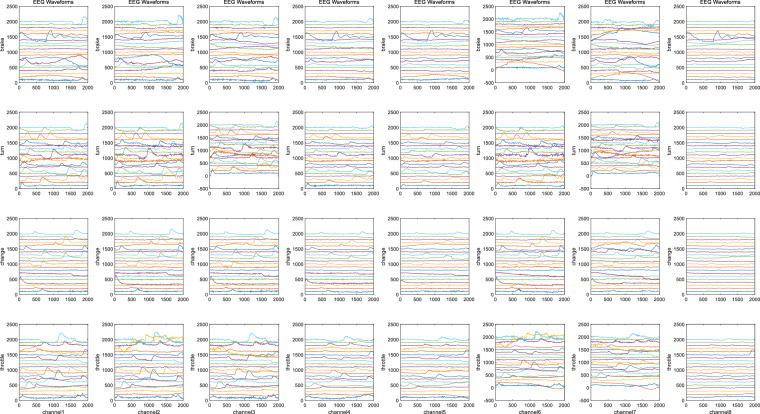
EEG signals of each epoch for 8 channels, 5 events, and 20 sampled subjects. Each row represents an event, and each column represents a channel. The waveform of each sample is stable, and most signals of the same event have the same trend without obvious abnormal fluctuation.

**Fig. 11:**
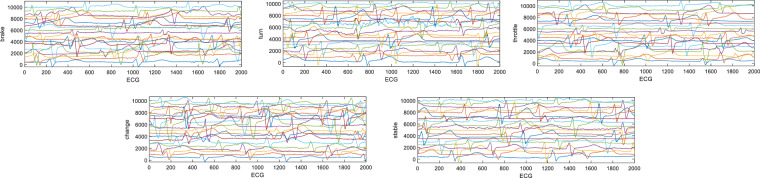
ECG signals of each epoch for all subjects. The waveform of the ECG shows a regular periodic peak, which indicates that the driving state is normal.

**Fig. 12:**
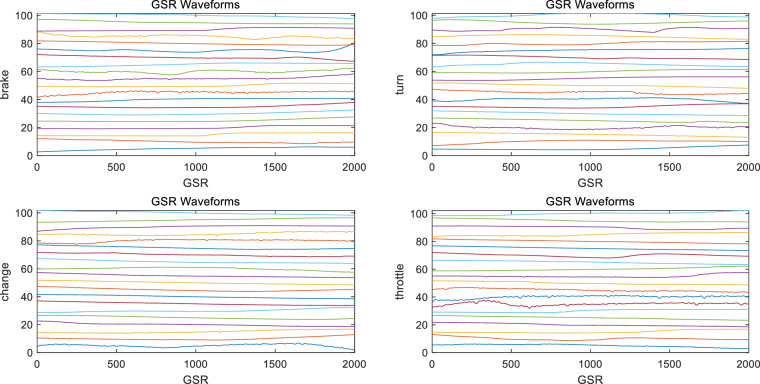
GSR signals of each epoch for all subjects. The GSR signals indicate the change in skin surface conductivity of the subjects.

**Fig. 13:**
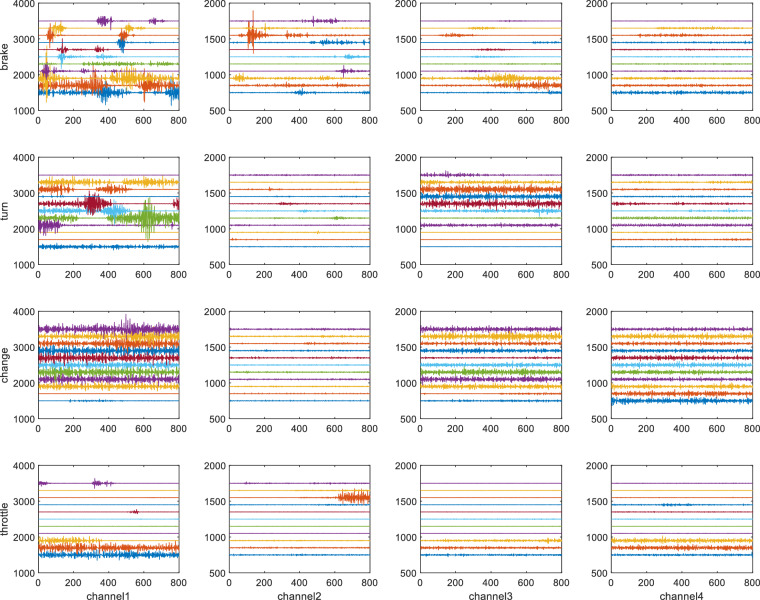
EMG signals of each epoch for all subjects and 4 channels. Channels 1,2-right calf, channels 3-4, arms. The signal response of channel 1 is the most obvious, which reflects the action of the subject when stepping on the pedal.

##### ECG signals

The ECG signal waveform of each epoch is shown in Fig. 11. For different subjects, the ECG of each subject must be normalized because the impedance conditions of the experiment may change. The heart rate in the figure is very normal and conforms to the parameter setting of the sensor.

##### GSR signals

The GSR signal waveform of each epoch is shown in Fig. 12. It can be seen that there is no obvious abnormal fluctuation in the GSR signal.

##### EMG signals

Figure 13 shows the waveform of EMG sigmals. When the muscles on the driver’s arms and legs produce actions, there will be an obvious fluctuation in the EMG waveform, such as when stepping on the brake or turning the steering wheel. The EMG signal has large noise interference, so we could extract enough obvious peaks as features.

The non-uniformity of fluctuations in EMG signals arises from the action potentials of different muscles during driving, e.g., the tibialis anterior muscle is more vigorous during pedal pressing and releasing, while the gastrocnemius muscle is relatively flat. For braking events, pressing the brake pedal urgently causes large observable fluctuations in EMG signals in the legs. Each individual also does not react and maneuver in the experiment in exactly the same way, which is one reason for the different EMG signals. In addition, the last two channels of EMG are placed on the arm, and the intensity of the arm muscle action is different from that of the leg.

The above results show that the driving condition of the subjects remains stable most of the time, without excessive stress and stress state during simulated driving. In particular, only under the stimulation of some emergency braking events involved in this case will the signals change substantially.

#### Correlation analysis of physiology and driving behaviours

**Fig. 14:**
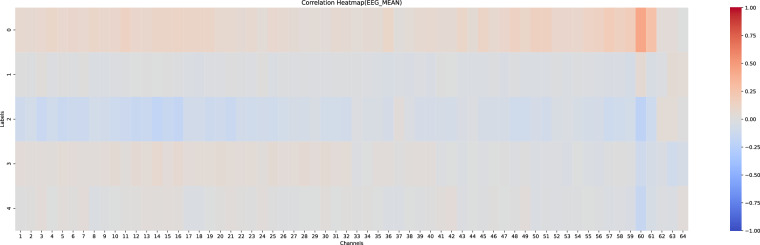
Correlation heatmap of mean values of physiological signals and five driving behaviors.

**Fig. 15:**
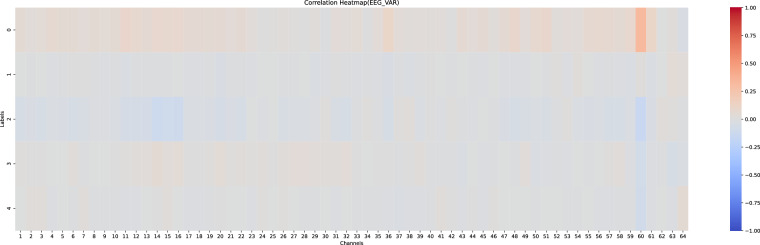
Correlation heatmap of variation values of physiological signals and five driving behaviors.

Figures 14, 15 illustrates the Spearman correlation analysis between the five driving behaviors and the mean and variation of 64-channel physiological signals (EEG, EMG, GSR), with the correlations shown as heat maps ranging from −1 to 1.

#### Eye tracking variable validation

**Fig. 16:**
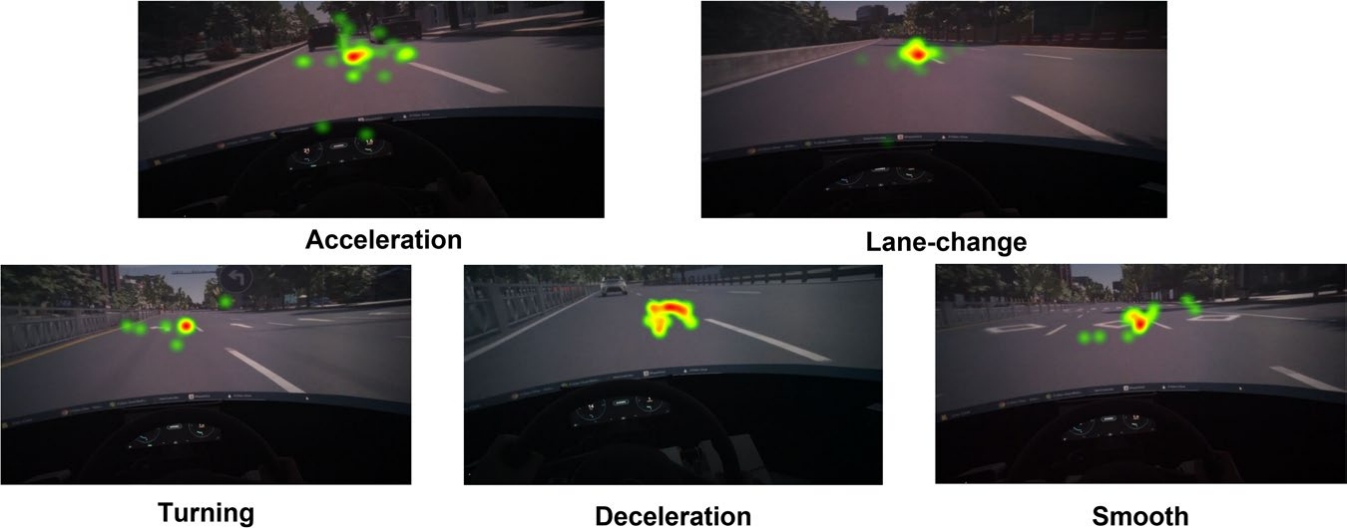
Subject 1’s Eye tracking: The scatter points are concentrated in the centre of the event, which indicates that the driving state is normal and without distraction.

The eye tracker recorded the *x* and *y* coordinates of the subject’s gaze at each point in time during the driving experiment, which can show the focus of subjects’ attention. As we introduced, the dataset mainly includes five categories of driving behaviours. In each behaviour, we found that the gazing patterns are notable at the main objects that induce driving events. Furthermore, the gazing pattern is also clear at the motor board, where speed and rpm are shown. This indicates that the subjects are sensitive to the car’s speed. To show the result, we plotted subject 1’s eye movement data scatter diagram of each event, which is shown in Fig. 16. The scatter diagram shows that the scatter points are concentrated in the centre of the event, and the driving state of the subjects in the experiment is normal and without distraction. Therefore, the data is effective.

### Validation of main vehicle parameters

#### Simulated driving environment

The simulated driving environment in this experiment is close to the actual vehicle environment. The hardware system of the simulator includes an adjustable real car seat, steering wheel, safety belt and shift lever. The software system of the simulator includes a model of an actual road in Beijing, dashboard, and a vehicle parameter recording system. Therefore, the physiological data collected during the simulated driving experiments can reflect the characteristics of actual driving to a certain extent.

#### Vehicle parameter verification

Vehicle parameters, such as speed, acceleration, accelerator pedal, brake pedal, engine speed, and gear position, are recorded by the simulator during the simulated driving test, which can directly reflect the driver’s behaviour. In the experiment, the subjects were asked to drive as smoothly as possible to avoid collisions to simulate the behaviour of real world driving as much as possible.

To ensure the repeatability of the experiment and the applicability of the dataset across subjects, we set the same route for each subject in the experiment under the same case, and the trigger time and content of events on the route were the same in different trials. Therefore, it can be concluded that the vehicle parameters of each subject in each case should show the same trend.

**Fig. 17:**
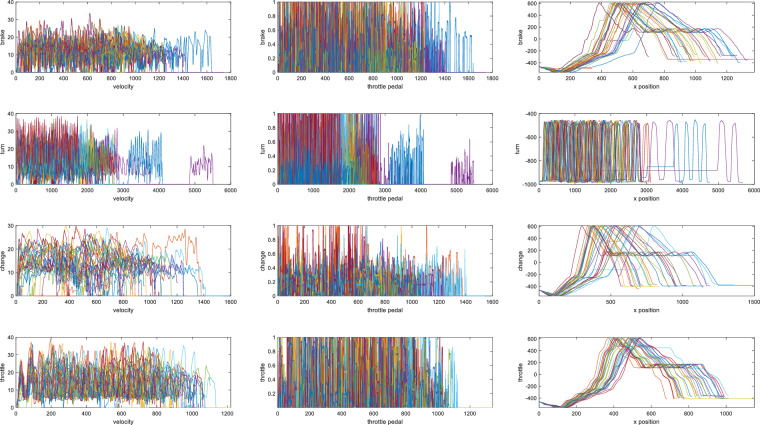
Vehicle parameters. (**a**) Velocity(m/s): the average speed is about 60 *km/h*, which is meeting the speed limit standard of urban roads with center lines. (**b**) Throttle pedal: for each driver, the acceleration signal is very strong at the beginning of driving, and then decreases until the vehicle speed approaches the speed limit. (**c**) *x*-position: when different subjects are driving, the change of vehicle position over time is almost the same, which ensures that the pre-designed events on the route are triggered in turn.

The curve of vehicle parameters changing with time is shown in Fig. 17. Each subfigure in Fig. 17 contains the curves of all 35 subjects, and each curve represents the time waveform of vehicle parameters during simulated driving. The figure shows that for each driving behaviour experiment, the trend of vehicle parameter data that changes with time has a strong similarity, which ensures the sameness of subject behaviour in each experiment. This shows that the behaviours of different subjects in the experiment are consistent, so the data obtained are repeated samples of five different types of behaviours. This ensures the repeatability of the sample and lays the foundation for subsequent analysis.

### Correlation validation of physiological data, behaviour and event labels

For this dataset, the most important point to prove is the correlation between physiological data and behaviour, that is, whether the physiological data contain the corresponding information of the driver’s behaviours. This section uses the collected data and the behaviour tags to build a model to illustrate this correlation.

#### Quality control of information interaction synchronization

Quality control of information interaction synchronization. The time of event labels and physiological data must be synchronized. In the above, we introduced a method of data synchronization. The software and hardware system of the 51Sim-One driving simulator writes the events into the event storage area of the Neuracle data acquisition software through serial ports according to the preset event determination conditions. For example, we set the judgement area at the point where the subject can just see the turning sign. At this time, it is deemed that the stimulus point of turning time has occurred, and this event will be marked in the system. Each event mark has a timestamp and is stored with the dataset. We use these event marks to cut the data to obtain event sample frames (epochs). Through experimental analysis, the delay of USB serial communication is very low (approximately 10 ms). Synchronization between data and marks can be ensured, which is also the basis of correlation analysis.

#### Classification of physiological data

Classification is a powerful illustration of data validity. We use the markers of vehicle parameters as data labels and physiological data as samples to train classifiers for classification tasks. If the data are valid, there should be commonalities between similar samples and differences between different samples. We first balanced the number of samples to ensure that the number for each category was roughly equal, divided the data into a training set, validation set and test set by 6:2:2^84^. Specifically, we specified stratify = y to ensure that the proportions of different classes in the training and test sets are the same as in the original dataset, and finally used the following two models for classification.

##### Linear discriminant analysis

Linear Discriminant Analysis (LDA)^83^ minimizes the distance between data of the same category and maximizes the distance between data of different categories through projection transformation of data; that is, it achieves the effect of dimension reduction and classification at the same time. To use LDA for the five classification tasks, the data must be reduced to no more than four dimensions. We combined EEG and EMG data, trained an LDA classifier to reduce each sample to four dimensions, and then verified the performance of the classifier. The overall classification accuracy of the model is 35.1%.

##### EEGNet

EEGNet is a compact EEG feature extraction convolution neural network with a deeply separable convolution structure. It has good generalization ability and performance in the case of limited data and can learn various interpretable features in a series of BCI tasks. After data preprocessing, we use EEGNet to classify the EEG epochs. We divided the training set and validation set for classification and used EEG data as samples and the mark generated by the simulator as labels. After all, the overall classification accuracy of the model is 49.8%. The results of the above two models show that the classification results of the two models are similar, which shows that there is indeed a correlation between samples and that it does not change with a change in the model.

The classification results show that the classification accuracy of the brake category is the highest, followed by throttle and changing_lanes, while the error rate of the turning and stable categories is higher. The reasons may be as follows:

1. For categories with high classification accuracy, throttle and brake involve the action of stepping on the accelerator and brake pedal, and their motion imagination characteristics may be quite obvious; thus, their sample characteristics are obvious, and the classification accuracy is much higher.
2. For categories with low classification accuracy, turning and stability, the main reason is the similarity and fuzziness between behaviours. Specifically, turning is easily misjudged as changing lanes because the driving actions of the two behaviours are similar, and the reason why the turn is misjudged as throttle may be that the accelerator pedal is pressed after turning; thus, the actions on the arm during turning and lane changing are not obvious.
3. The stable category that we designed to work as the control group tends to be misjudged as many other categories, especially turning, changing lanes and throttle, possibly because the throttle pedal is pressed under normal driving conditions, and the differences are not obvious.

#### Validation of multimodal data

To improve the resolution of EEG signals, we use multimodal data(e.g. combining EEG with EMG and ECG) as an assistant. Multimodal data actually promote EEG data to a higher dimension. Although compared with the 59 effective channels of EEG signals, EMG has only 4 channels, the behavioural information provided by EMG is crucial.

##### Multimodal physiological Net(MMPNet)

**Fig. 18:**
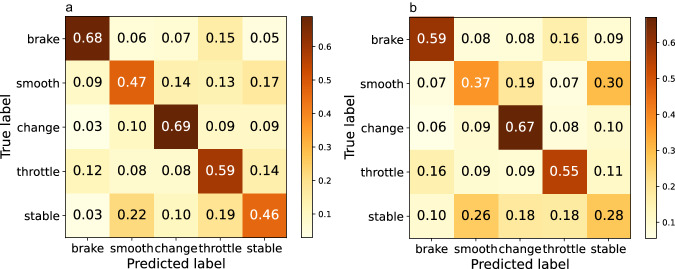
The performance comparison of MMPNet model between multimodal data and EEG data shows that the classification accuracy of multimodal data is significantly improved compared with that of single mode data (*p* = 1.2956 × 10^−9^). (**a**) Multimodal data. (**b**) EEG Only.

MMPNet is a neural network model specially designed for multimodal physiological data, and its structure is shown in Fig. 2. The overall classification accuracy of the MMPNet model under multimodal data is 62.6%, while the accuracy of MMPNet model using EEG only is 55.7%, and the confusion matrices are shown in Fig. 18.

##### EEGNet

The overall classification accuracy of EEGNet model under multimodal data is 55%, which is significantly improved compared with 49.8% of the single-mode data.

Overall, the classification accuracy of several models is shown in Table 6. It can be seen that the classification performance of MMPNet exceeds that of the baseline model EEGNet. The performance comparison of MMPNet model between multimodal data and EEG data shows that the classification accuracy of multimodal data is significantly improved compared with that of single mode data (*p* = 1.2956 × 10^−9^).

**Table 6:** Driving behavior classification accuracy of several validation models.

Data	Models				
	LDA	EEGNet	LSTM	CNN	MMPNet
Only EEG	33.5%	49.8%	58.3%	56.2%	55.7%
Multimodal Data	36.9%	57.7%	66.2%	62.5%	65.3%

The results show that the accuracy of the model is improved to a certain extent by multimodal data. Compared with the situation where only single mode data are used, EMG provides information on the driver’s arm and leg movements, which makes it easy to distinguish between the three behaviours of acceleration, turning and stability that were easily confused before due to the differences in pedal and steering wheel movements.

**Fig. 19:**
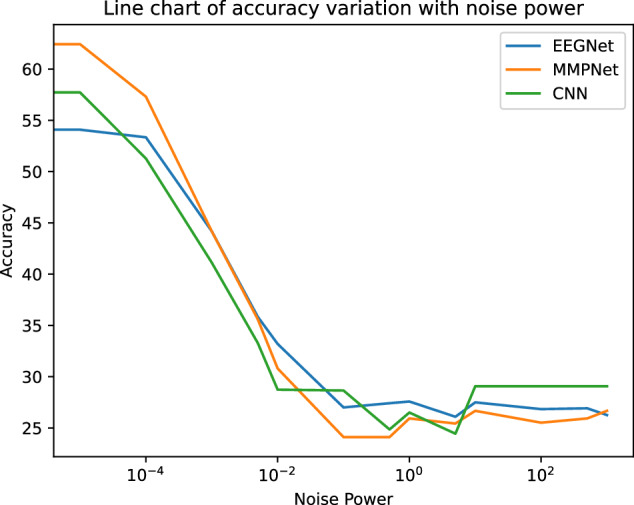
Line chart of accuracy variation with noise power.

We adopt a strategy to demonstrate the effectiveness of the above classification results, which involves adding additive Gaussian noise with a mean of zero and a gradually increasing variance to the data. Figure 19 illustrates the variation of the performance of the three models for different noise powers. It can be seen that as the variance of additive Gaussian noise increases, the classification performance will deteriorate. It can be seen that when the power of noise exceeds a certain limit, the classification results of the model will be very close to completely random. Hence, we can clearly see that the physiological data has strong separability, which reflects the high quality of the data.

## Usage Notes

The original data and preprocessed data of multimodal physiological signals can be downloaded from Figshare. Users interested in the dataset can register on the website and download the dataset locally. The original dataset and the preprocessed dataset are named as “Driving behaviour multimodal human factors original dataset” and “Driving behaviour multimodal human factors preprocessed dataset”, respectively.

After the dataset is downloaded, users can process EEG through MATLAB’s EEGLAB plug-in. We recommend that researchers use EEGLAB version 2021 and MATLAB R2021b on Windows 10 or Linux. EEGLAB can help complete EEG preprocessing steps such as filtering, segmentation and ICA. The code for batch preprocessing of EEG signals will also be provided in “Code availability”. The file formats of EMG, GSR and ECG signals are consistent with those of the EEG signals, which can also be imported and processed through MATLAB. The batch preprocessing codes of EMG, GSR and ECG signals will also be provided in “Code availability”.

Additionally, we suggest the following data processing steps:

- Download the dataset from the above website and save it locally. Record the save path.
- Check whether the dataset is complete. Each raw data point consists of two parts, namely “data. bdf” and “evt. bdf”.
- Import the EEGLAB plug-in to MATLAB and load the dataset.
- Complete data preprocessing, including but not limited to filtering and segmentation.
- Further analysis and research can be performed using the preprocessed data.

## Data Availability

Readers can access the tutorials and code of our original and preprocessed datasets on Github (https://github.com/zwqzwq0/MPDB). Two folders called preprocessing and classification can be found, which contain MATLAB code for preprocessing and python code for classification.
